# Preliminary Studies Demonstrating Acetoclastic Methanogenesis in a Rat Colonic Ring Model

**DOI:** 10.1155/2013/540967

**Published:** 2013-07-15

**Authors:** Edward A. Carter, Ronald G. Barr

**Affiliations:** ^1^Departments of Pediatrics, Massachusetts General Hospital, Harvard Medical School, Boston, MA, USA; ^2^Montreal Children's Hospital Research Institute, McGill University, Montreal, QC, Canada; ^3^Pediatric Gastrointestinal Unit, Massachusetts General Hospital, 114 16th Street (114-3503), Charlestown, MA 02129-4004, USA

## Abstract

Washed rat colonic rings were incubated in closed flasks under N_2_ at physiologic pH and temperature levels. In the absence of an exogenous substrate, negligible H_2_ but some CH_4_ concentrations were detected in vitro after one hour of incubation, but high concentrations (H_2_ > 100 ppm, CH_4_ > 10 ppm) of both gases were found after 24 hours of culture. Production of H_2_ and CH_4_ by the washed colonic rings was stimulated by lactose addition. Maximum H_2_ production occurred at about pH 7.0, while maximum CH_4_ production occurred between pH 4.0 and 6.0. The increased production of both gases at 24 hours was associated with dramatic increases (10^4^-fold) in anaerobic bacteria colony counts on the colonic rings and in the incubation media, as well as dramatic increases (100-fold) in acetate concentrations in the media, while lactate concentrations first rose and then fell significantly. These results suggest that gas production in colonic ring preparations is subject to quantitative changes in microbiota, pH, and metabolite formation analogous to in vivo conditions. In addition, microbiota firmly attached to colonic tissue appears to utilize colonic tissue to support its growth in the absence of an exogenous substrate.

## 1. Introduction

Humans and animal breath contains gaseous materials not found in normal air; these materials are thought to be products of bacterial and nonbacterial metabolism [1–3]. Two of these gases, hydrogen (H_2_) and methane (CH_4_), have attracted considerable attention in recent years, [4, 5] including a study suggesting that obese patients who have methane in their breath have significantly higher body mass indexes which may play a role in obesity [6–8]. Breath H_2_ has been shown (a) to be reduced with fasting, (b) to be responsive to exogenous substrate ingestion, (c) to vary with the various populations, (d) to become elevated in some disease states, (e) to be subjected to lung excretion capacity, (f) to be dependent on the presence of bacteria, and (g) to show a close response to colonic bacterial content. By contrast, breath CH_4_ appears (a) to be unresponsive to carbohydrate substrate ingestion in adults (but not necessarily in infants), (b) to be produced at a constant rate, (c) to not be present universally in all populations, (d) to be elevated in disease states (especially cancer), and (e) to be produced by the distal colonic bacteria.

Considerable attention has been devoted toward clinical applications for the measurement of breath H_2_ and CH_4_ levels. These studies have focused on H_2_ and CH_4_ generation in vivo by human or animal models or have used in vitro assays to monitor H_2_ and CH_4_ production using stool (feces) homogenates. Although the ultimate applicability of these breath tests have been improved, intrinsic limits exist to a complete understanding of the molecular mechanisms involved in the actual production of the gases at the site of origin, that is, the colon. In vivo fecal homogenate assays have clearly advanced our understanding of which substrates function in the production of which gases. However, these systems represent studies on bacterial populations that are “distal to” and “outside of” the colon. The present communication details an initial attempt to develop a model system to monitor the production of gaseous products in vitro using the bacteria adherent to the colon itself.

## 2. Materials and Methods

### 2.1. Animals

Female rats weighing 125–175 g (CD, Charles River Breeding Farms, Wilmington, MA, USA) or germ-free female rats of similar weight (Taconic Farms, NY, USA) were used throughout these studies and maintained in accordance with the guidelines of the Committee on Animals of the Harvard Medical School and those prepared by the Committee on Care and Use of Laboratory Animals, the Institute of Laboratory Animal Resources, National Research Council (DHEW, Publication No. NIH 78-23, Revised 1978). All animals were fasted overnight prior to use. Typically, six animals were used in each experiment.

### 2.2. Colonic Ring Preparation

Rats were sacrificed by cervical dislocation. The colon was removed, washed with 50 mL sterile saline, and then cut into 2-3 cm rings. All subsequent work was done inside an anaerobic chamber (Coy Laboratory Products, Grass Lake, MI, USA). One gram of tissue was incubated in 10 mL sterile buffer (100 mM Tris buffer, pH 7.4, 33 mM potassium chloride, 120 mM sodium chloride) in a sterile 50 mL Erlenmeyer flask. The contents of the flasks were gassed for 30 sec with N_2_ (10 liter/min), after which the flask was capped with a rubber stopper and incubated at 37°C in a shaking water bath (100 strokes/min). At prescribed intervals, the flasks were removed from the water bath. A 50 cc syringe filled with sterile water was attached to a 22-gauge needle and inserted through the rubber stopper. An empty 50 cc syringe was attached to a three-way stopcock which was attached to a needle and also inserted through the rubber stopper. The water filled syringe was then emptied, expelling the gas into the empty syringe. The gas-filled syringe was closed and removed from the flask after about 40 mL of gas was collected. The needle was removed and a 0.45 *μ*m sterile filter unit (Millipore Millex) was attached. A new needle was attached to the filter unit and inserted into a sterile vacutainer. The stopcock was opened and the gas passed into the vacutainer.

### 2.3. Fecal Preparations

Fecal material (formed pellets) was taken from the colon of rats immediately after sacrifice and handled in the anaerobic chamber as described previously. The fecal material was homogenized (1 gram/10 mL) in a Waring blender for 1 min in the media described previously. Ten milliliters of homogenate was placed in a sterile 50 mL Erlenmeyer flask and gassed for 30 sec with N_2_ (10 liter/min). The flasks were then capped and incubated at 37°C in a shaking water bath.

### 2.4. H_2_ and CH_4_ Analysis

The gaseous samples were analyzed for H_2_ and CH_4_ by gas chromatographic procedures described previously [1].

### 2.5. Colony Counts

Bacterial colony units were determined in both the bathing media and on the tissue using horse blood agar plates. Aliquots of media were serially diluted with sterile PBS. Aliquots (100 *λ*) were then transferred to the horse agar plates and anaerobically incubated at 37°C for 18 hours. Tissue was decanted from the flasks and lightly patted on sponges to remove excess media. Subsequently, the tissue samples were homogenized in 10 mL sterile PBS and serially diluted with additional sterile PBS. Again, 100 *λ* aliquots were transferred to the horse agar plates and anaerobically incubated at 37°C for 18 hours.

### 2.6. Acetate and Lactate Measurements

Acetate and lactate concentrations in the media were determined in the bathing media using Boehringer-Mannheim kits (West Germany). For these experiments the colonic rings and media were transferred to 15 mL plastic tubes, sealed and spun at 100 ×g for 15 min at room temperature. The supernatant was deproteinized using perchloric acid for lactate and charcoal and boiling water for acetate.

### 2.7. Statistical Analysis

Analyses were performed by standard Student's unpaired *T* test.

## 3. Results

We first examined the ability of fecal homogenates made from the colonic fecal contents to produce H_2_ and CH_4_ with and without lactose addition after 1 or 24 hr of incubation; these two time points were chosen because human fecal homogenates have been shown to produce H_2_ or CH_4_ after 1 or 24 hr of incubation with lactose or glycoprotein, respectively [9]. As can be seen in Table 1, homogenates made from fecal material taken from the colon produced significant amounts of H_2_. Significantly more (*P* < 0.01) H_2_ was produced by the fecal colonic homogenates with lactose addition after either 1 or 24 hr incubation. In addition, the colonic fecal homogenates generated CH_4_, a production that was increased with time (24 hr of incubation), but not with lactose addition (Table 1). No H_2_ or CH_4_ was detected in flasks incubated with the sterilized media alone.

**Figure 1:**
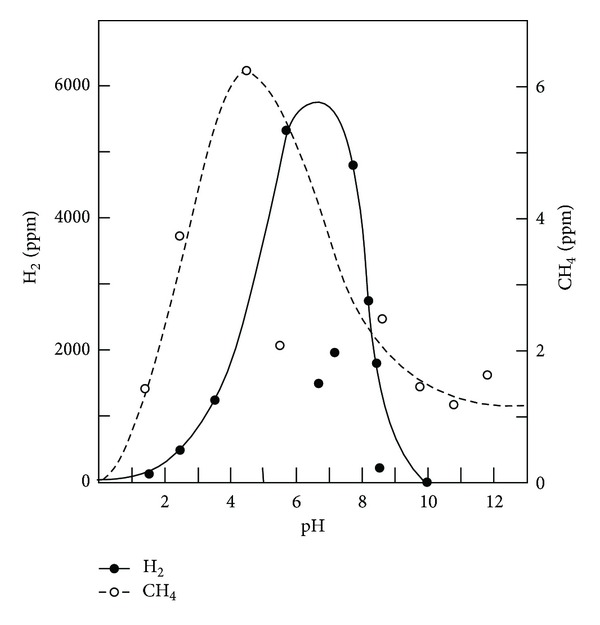
pH dependency of H_2_ and CH_4_ production after incubation of washed rat colonic rings for 24 hours. Washed rat colonic rings were incubated and H_2_ or CH_4_ was assayed in the air space above the incubation mixture, as described in the text. The results are the mean of 2-3 experiments run at each pH.

We next examined the ability of washed colonic rings to produce H_2_ and CH_4_ in the presence or absence of lactose at 1 and 24 hr incubation. As can be seen in Table 2, negligible amounts of H_2_ were detected with or without lactose after 1 hour of incubation, although small amounts of CH_4_ were detected. However, copious amounts of H_2_ and CH_4_ were detected after 24 hr of incubation. The addition of lactose increased H_2_ and CH_4_ production significantly at 24 hr (*P* < 0.01). Colonic preparations from germ-free rats incubated in the sterilized media did not produce H_2_ or CH_4_ in the absence or presence of lactose. This confirms a previous report that germ-free rats produce no methane in vivo [10]. The pH for maximum H_2_ production at 24 hr appeared to be 7.0, while the pH for maximum CH_4_ production was closer to 5 (Figure 1).

**Figure 2:**
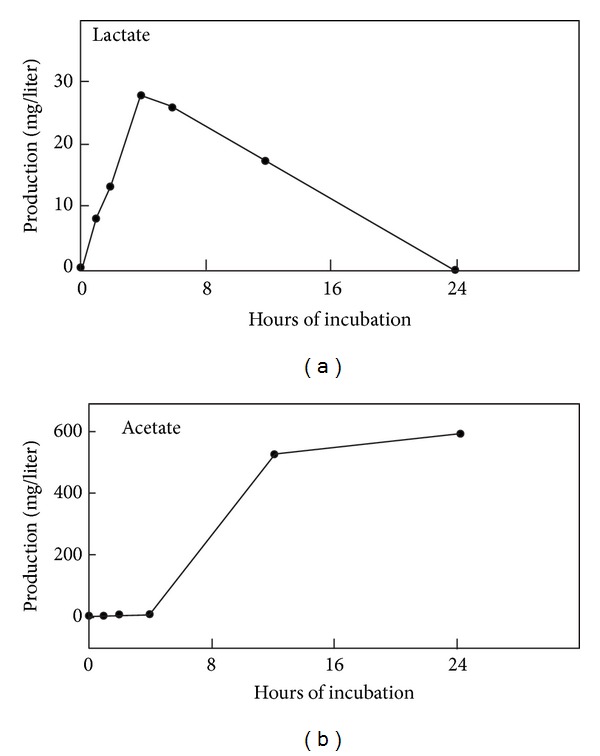
Lactate and acetate production by washed rat colonic rings. Washed rat colonic rings were incubated, and lactate or acetate concentrations were determined in the incubation mixture, as described in the text. The results are the mean of two experiments at each time point.

We next attempted to correlate the increases in H_2_ and CH_4_ production in the absence of lactose with actual bacterial colony counts as well as lactate and acetate concentrations, known metabolites of ruminant bacterial digestion [11]. As can be seen in Table 3, the colony counts observed in the media or on the colonic tissue increased some 10^4^-fold after 24 hr of incubation. Lactate concentrations appeared to drop dramatically after 24 hr of incubation but acetate concentrations increased 100-fold (Table 3). To confirm these observations and to determine the time course, studies were repeated with determination of lactate and acetate at 1, 2, 3, 4, 6, 12, and 24 hours. As can be seen in Figure 2, lactate concentrations gradually increased during the first 4 hr of incubation, but fell off rapidly after 6 hr. By contrast, acetate concentrations began to increase after 6 hrs of incubation and increased to levels 100 times those seen at one hour.

## 4. Discussion

Methanogens are microorganisms that produce methane as a metabolic byproduct in anoxic conditions. They are common in the guts of animals such as ruminants and humans, where they are responsible for the methane content of belching in ruminants and flatulence in humans [12]. Methanogens have been found to be present in the colons of rats [10]. Rodkey et al. [8] reported CH_4_ production rates of as high as 29 mL/day with Sprague-Dawley rats. In a similar system of in vivo gas collection, we have observed both H_2_ and CH_4_ production in vivo by the rats in our facility (Carter, unpublished observations). The washed colonic tissue from the rats in our study produced both CH_4_ and acetate. This may be related to acetoclastic methanogenesis (reaction 6, Table 2 [13]) based on the increase in the methane production by 400% under the same conditions.

It is generally felt that obesity disorders are the result, in part, of the gut microbiota which contributes to the energy imbalance because of its involvement in energy intake, conversion, and storage. Culture-independent methods have showed that high proportions of methanogens can comprise up to 10% of all anaerobes in the colons of healthy adults [14]. The development of *Methanobrevibacter smithii* in anorexic nervosa patients may be associated with an adaptive attempt towards optimal exploitation of the low caloric diet of anorexic patients [14]. Hence, an increase in *M. smithii* leads to the optimization of food transformation in low caloric diets. *M. smithii* could also be related to constipation, a common condition for anorexic patients [14].

It has been proposed that the role of *Methanobrevibacter smithii* in weight gain in animals is related to the ability of the *M smithii *to scavenge hydrogen produced by syntrophic organisms for its hydrogen-requiring anaerobic metabolism, producing methane as a byproduct [7]. These authors proposed that the scavenging of hydrogen allows the syntrophic organisms to be more productive, increasing short chain fatty acids (SCFA) production and availability of calories for the host [7]. In their study [7], the presence of hydrogen and methane on breath test, but not either methane or hydrogen alone, was associated with higher BMI and percent body fat. The authors postulated that, in the subjects that had an abundance of hydrogen to fuel methane production, the intestinal methanogens could also contribute to enhanced energy harvest. These authors previously noted an association between breath methane and constipation in human subjects and, using an in vivo animal model, demonstrated that methane gas directly slows transit in the gut by 59% [7]. The authors hypothesized that the slowing of transit could result in greater time to harvest nutrients and absorption of calories, representing another potential mechanism for weight gain [7].

In summary, the present report details our initial studies to develop an in vitro model for monitoring H_2_ and CH_4_ production that will allow closer examination of the association of the evolution of these gases with changes in the tissue microbiota involved, particularly the methanogens, adherent to colonic tissue. Such a model may prove useful in the elucidation of the molecular mechanism(s) involved in the elaboration of these gases and the role of these microorganisms in the development of obesity.

## Figures and Tables

**Table 1:** H_2_ and CH_4_ production by rat fecal homogenates.

Gas production (ppm)^a^				
	H_2_		CH_4_	
	1 hour	24 hours	1 hour	24 hours
Without added lactose	486 ± 220	380 ± 415	17 ± 15	56 ± 37
With added lactose	2,001 ± 706	14,235 ± 17,004	48 ± 12	144 ± 42

^a^One gram of freshly collected feces in the colon was incubated as described in Methods. Lactose (1.25 g% final concentration) was added where indicated. H_2_ and CH_4_ were determined as described previously [1]. The concentration of the gases is expressed as part per million. The results are the average of three experiments, mean ± SD, with six rats in each experiment.

**Table 2:** H_2_ and CH_4_ production by rat colonic rings.

Gas production (ppm)^a^				
	H_2_		CH_4_	
	1 hour	24 hours	1 hour	24 hours
Without added lactose	0	2,280 ± 1,695	0.7 ± 0.3	1.5 ± 0.4
With added lactose	0	34,280 ± 8,927	0.8 ± 0.1	6.3 ± 0.6

^a^One gram of washed rat colonic rings was incubated as described in Methods. Lactose (1.25 g% final concentration) was added where indicated. H_2_ and CH_4_ were determined as described previously [1]. The concentration of the gases is expressed as part per million. The results are the average of three experiments, mean ± SD, with six rats in each experiment.

**Table 3:** Bacterial counts, lactate, and acetate concentrations in rat colonic ring preparations.

Assayed system	Concentrations	
	1 hour (*N* = 3, mean ± SD)	24 hours (*N* = 3, mean ± SD)
Bacterial counts^a^		
Tissue	4.0 ± 0.4 × 10^5^	5.0 ± 5.0 × 10^9^
Media	5.0 ± 0.5 × 10^5^	6.0 ± 6.0 × 10^9^
Lactate^b^	20.0 ± 1.0	3.0 ± 1.0
Acetate^b^	5.0 ± 1.0	693 ± 1.5

^a^Washed rat colonic rings were incubated at the times indicated, as described in Methods.

Bacterial counts expressed as counts per gram of tissue or per 10 mL of media.

^b^Lactate or acetate concentrations were determined in the media, as described in Methods, and are expressed in mg/liter of media. The results are the average of three experiments, mean ± SD, with six rats in each experiment.
